# Research on Improving NVH Performance of Automobile Side Door Glass in Use Based on 6 Sigma Method

**DOI:** 10.3390/ma14133748

**Published:** 2021-07-04

**Authors:** Chunlong Ma, Dongyan Shi, Mengnan Wang, Dongze He, Chao Li, Xingsheng Yu

**Affiliations:** 1 College of Mechanical and Electrical Engineering, Harbin Engineering University, Harbin 150001, China; machunlong@hrbeu.edu.cn (C.M.); shidongyan@hrbeu.edu.cn (D.S.); hdz2012071506@126.com (D.H.); lichao327@hrbeu.edu.cn (C.L.); 2 Department of Automotive, Harbin Vocational & Technical College, Harbin 150000, China; 257_hrbeu_edu@sina.com

**Keywords:** squeak and rattle of automobile side door glass, dimension analysis of 6 Sigma, instability, squeak noise, numerical simulation

## Abstract

Automobile side door glass squeaks and rattles during use. This abnormal noise can make the driver and occupants irritable and reduce the comfort of the automobile. This reduces the sales of this automobile. This paper analyzes and determines the cause of squeak and rattle during the lifting and lowering process of the side door glass of an automobile. The noise is due to four reasons. One is that the distance between the inner waterproof belt and the automobile side door glass of the automobile is unreasonable, causing excessive friction between the automobile side door glass and the inner waterproof belt during the automobile side door glass up and down movement. Other factors affecting squeak and rattle may be the distance between the automobile side door sheet metal and the automobile side door glass, the thickness of the automobile side door glass and the characteristics of the inner waterproof belt. The first two dimensions are analyzed using the 6 sigma method, and the structure of the inner waterproof belt is improved and the flocking position is adjusted. The squeak and rattle phenomenon is explained using the implicit dynamic analysis method ABAQUS, and the compression load deflection after the installation of the inner waterproof belt is 3–9 N/100 mm. This research completely solves the squeak and rattle problem caused by the up and down movement of the side door glass of the automobile. This research has significance for solving practical engineering problems.

## 1. Introduction

The automobile door glass is a part of the automobile glass system, which moves up or down along the glass guide groove under the drive of the glass lifter. As the degree of electrification of automobiles continues to increase, electric lift door glasses have become standard equipment in automobiles. Because the lift glass of automobile doors is used frequently, and the door glass is close to the driver and passengers, users hope that the noise generated by the door glass can be effectively controlled during the process of lifting the door glass. In other words, users hope that the sound quality of the door glass system during use will be better. So-called sound quality is an objective description of people’s subjective feelings of sound, and it is an important factor that affects the competitiveness of automotive products [1].

To solve this problem, scholars at home and abroad have performed extensive research. There is a consensus in academic circles that the friction vibration is caused by the negative slope of the friction coefficient–relative sliding velocity curve [2,3]. Ma [4,5] performed relevant research on the lifting and lowering of the door. To establish the phenomenology of friction noise, many experiments have been carried out. Yokoi and Nakai [6] performed an experiment with a rod–disk contact and studied friction noise on various random surfaces. Boyko L. Stoimenov [7] used two rectangular stainless steel plates with similar surface roughness under dry friction conditions. The effect of surface roughness on the frequency of squeak friction sound in dry friction contact was clarified. M.O. Othman [8,9] carried out a series of experiments on noise related to surface roughness, and established a relationship between the sound pressure level and the surface roughness under different contact loads. Ben Abdelounis H [10] studied the friction noise between dry surfaces, and concluded that dry contact and roughness under light pressure were the main causes of the noise. Alain Le Bot E [11] explored the frictional noise of rough surfaces in contact with a light load. It was found that the main mechanism of sound generation was the normal vibration of the surface caused by the impact between opposing rough bodies, and the friction noise is dependent on the contact area of the rough surface.

A study by Ben Abdelounis [12] showed that sliding solids are almost uncoupled under the conditions of a normal light load and a rough surface, so contact does not change the natural frequency of the sliding solid. Ben Abdelounis [13] used ABAQUS to simulate friction noise. The results showed that roughness plays an important role in noise. Zhen yu Yang [14] used lubrication to reduce noise in material friction. Wang lin’s [15] research found that different types of lubricants have different effects on improving noise and stick–slip phenomena of acrylonitrile butadiene styrene (ABS) materials. Friction noise and stick–slip of ABS decrease with increasing lubricant content. Jaehyeon Nam [16] studied the friction noise by applying lubricant on the friction surface after cleaning the contact surface compare to without any surface treatment. It was found that the friction coefficient of the contact surface can be greatly reduced, and the friction noise can be effectively prevented by using lubricant.

The 6 sigma approach focuses on the voice of the customer and basically achieves zero error. The design of the 6 sigma approach focuses on improving product quality and meeting customer expectations by adjusting nominal values of controllable design variables and optimizing tolerances without increasing costs. Yuqiang L [17] introduced the 6 sigma optimization design process. Its first five stages correspond to the definition, measure, analysis, improvement, and control stages. Krehbiel, T.C implemented a 6 sigma program for the subsidiary of a fortune 100 company to improve its quarterly financial reporting process, and 6 sigma can also be used to save financial costs [18]. Koo Il Seob [19] used the 6 sigma method to analyze the influence of internal factors on customer satisfaction.

**Figure 1:**
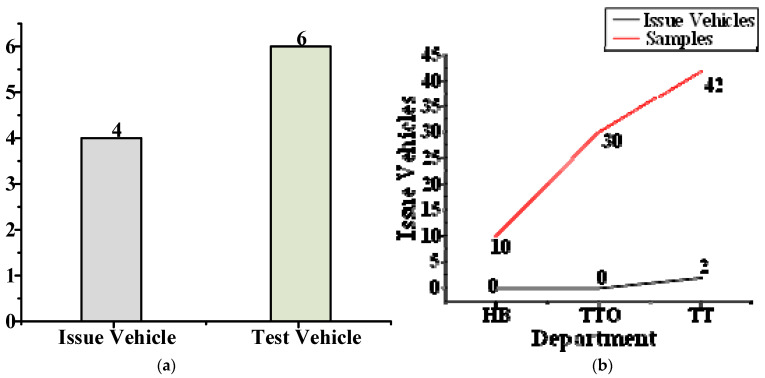
Vehicle survey map. (**a**) Statistical chart of problem vehicles; (**b**) number of problem vehicles by department. HB is Hard Tooled Functional Build. TTO is Tool Try-Out. TT is Tooling Trial. HB, TTO and TT are the three departments in automobile production.

The windows of some automobiles will appear to squeak and rattle (S&R) when it goes down, although this only occurs in some automobile models. To solve the problem of S&R when the window goes down, a sample survey was carried out on the vehicles in each stage of production, as shown in Figure 1b. Four automobiles had window problems, as shown in Figure 1a. The front windows of three automobiles had the problem and only one automobile’s rear window had the problem.

**Figure 2:**
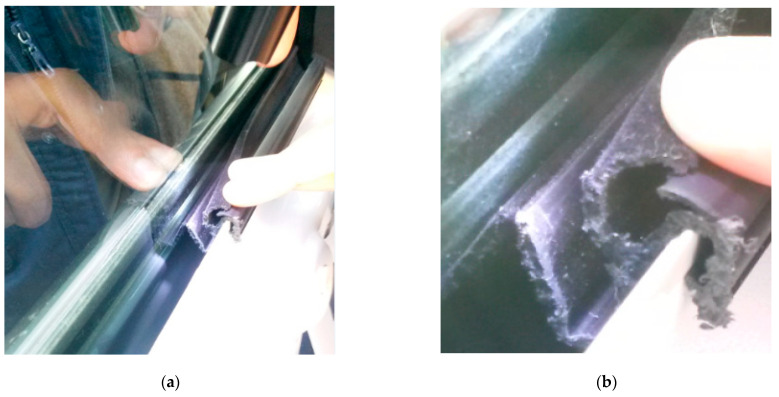
Schematic diagram of window and inner belt. (**a**) Schematic diagram of the window and inner belt; (**b**) physical picture of inner belt.

After repeated experiments, it was determined that there were two kinds of rattle noise environment: (1) when the window goes down from the top to the bottom; and (2) low humidity (20–50%). Figure 2a shows the position of the window glass in relation to the inner belt. Figure 2b is a physical image of the inner belt.

In this paper, the 6 sigma analysis method was used to analyze the noise problem of a motor vehicle’s window when it drops. According to the analysis steps, the key factors that may affect noise were measured and checked one by one, and the cause of the noise was found: the friction between the falling window glass and the inner belt. After that, numerical simulation was carried out to solve the problem, and the characteristics of the inner belt in the window were improved to solve the noise problem.

The highlights of this article are: ➀ the use of the 6 Sigma method to solve practical engineering problems; ➁ the use of fishbone diagrams to analyze possible problems, and the use of experimental equipment for verification; ➂ the use of the finite element method to help analyze and verify conclusions.

## 2. Experimental Device

### 2.1. Inner Belt

#### 2.1.1. Material Properties

**Figure 3:**
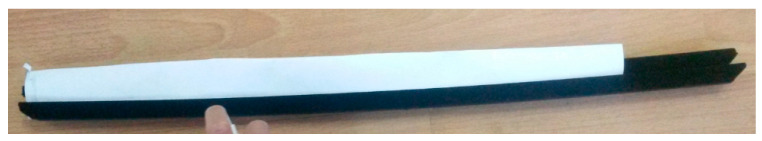
Inner belt. The inner belt was removed from a window with S&R.

The inner belt of the automobile is made of rubber. The performance of the rubber directly determines the performance of the inner belt. The performance of the rubber used in the inner belt is shown in Table 1. The inner belt used in this experiment is shown in Figure 3.

#### 2.1.2. Section Characteristics

**Figure 4:**
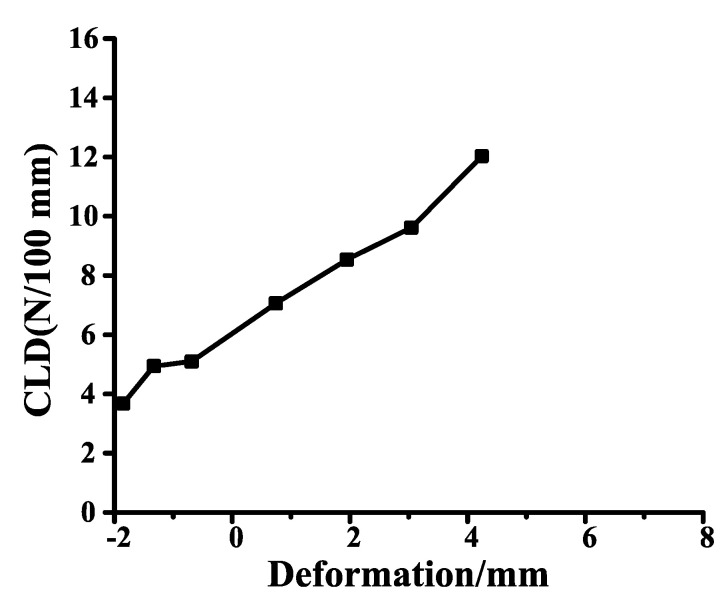
Section characteristics of inner belt. The Y axis represents CLD, the X axis represents deformation, and 0 mm is the standard used.

The compression load deflection (CLD) of the inner belt is not only determined by the tolerance fit, but also by the section characteristics of the inner belt. The default unit of CLD is N/100 mm. The section characteristics refer to the curve of the relationship between the CLD of the inner belt and the amount of compression, as shown in Figure 4.

In the deformation curve of the inner belt, the scale of abscissa is after matching, that is to say, 0 is the standard scale, and CLD should be between 4–6 N/100 mm after inner belt installation.

### 2.2. Window Side Door Point Measuring Tool

**Figure 5:**
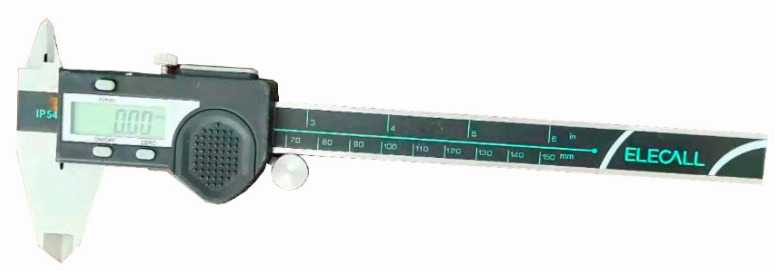
Vernier caliper. The resolution of the vernier caliper is 0.01 mm.

The main purpose of this experiment is to explore the sealing of the inner belt. The biggest influence on the sealing is the matching size of the inner belt and the window. To explore the relationship between the two, it is necessary to measure and analyze the key dimensions of car windows. Figure 5 shows the main measuring tool of this experiment. The resolution of the vernier caliper can reach 0.01 mm.

### 2.3. MR3-800 Coating

**Figure 6:**
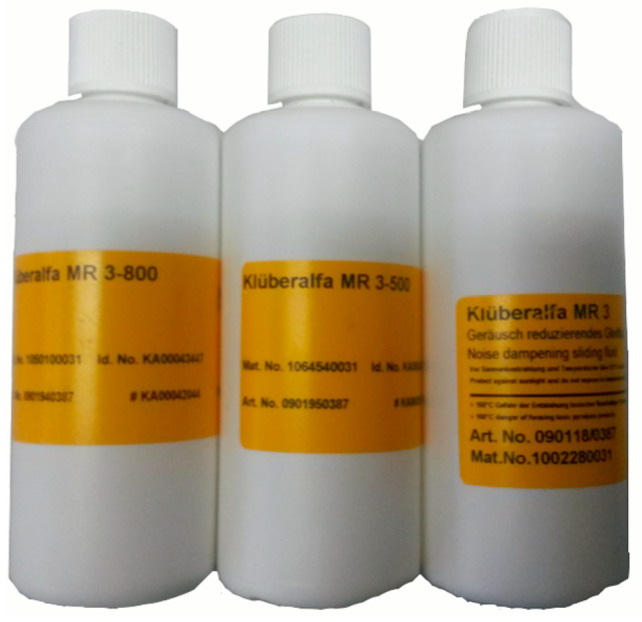
MR3-800. The friction coefficient can be reduced effectively after MR3-800 application.

*MR*3-800 coating is a sliding fluid sealing lubricant, as shown in Figure 6, which can reduce the friction between glass and inner belt, and can effectively prevent the “squeak” sound in a short time. In addition, due to the good compatibility of the MR3-800 coating, the MR3-800 coating can be used in combination with most materials (including leather, textiles, sealing rings or plastics) on the interior or body of the automobile. The MR3-800 coating can quickly form an oil film after use.

### 2.4. Motor and Transmission Mechanism

**Figure 7:**
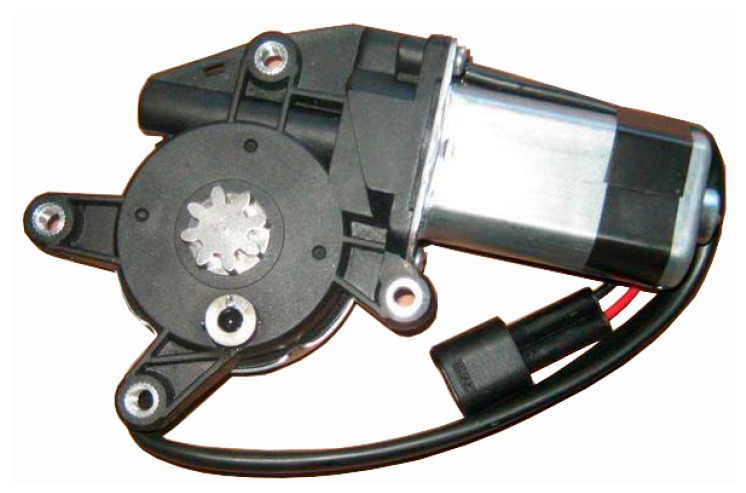
Motor and transmission mechanism. The movement of the experimental glass is driven by the mechanism.

The lifter used for the door is an electric rope wheel lifter, which can generate rotary motion under the drag of the direct current motor. The rotary motion is transformed into low-speed linear motion (about 20 mm/s) through a worm and rope wheel to further drive the side window glass to lift. The structure of the electric motor and transmission mechanism is shown in Figure 7.

### 2.5. Coordination between Glass and Inner Belt

**Figure 8:**
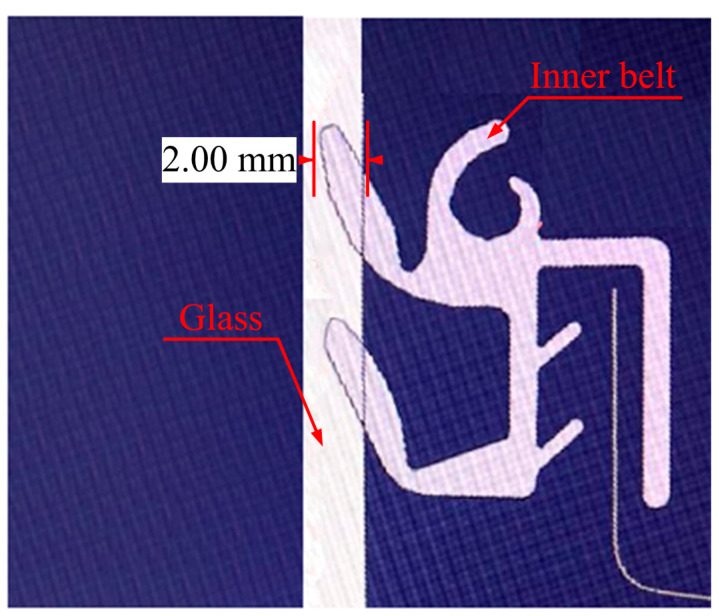
The interference principle diagram of the inner belt and glass.

The motor drives the car side window glass to move up and down. Because there is a certain amount of interference between the car glass and the inner belt, which is about 2 mm, as shown in Figure 8, there is a certain CLD after the installation of the inner belt. Driven by the motor and transmission mechanism, the side window of the automobile is moved up and down to explore the source of the “rattle” sound when the side window of the car goes down. In the following chapters, an improved inner belt will be tested.

## 3. Analysis of S&R Problem Based on the 6 Sigma Method

The 6 sigma method is one of the analysis methods that can be used for problems with an unknown cause. The 6 Sigma method is divided into five steps: definition, measurement, analysis, improvement and control. This paper is a scientific article, but control is biased towards cost and profit, so control is used for verification in the following chapters.

**Figure 9:**
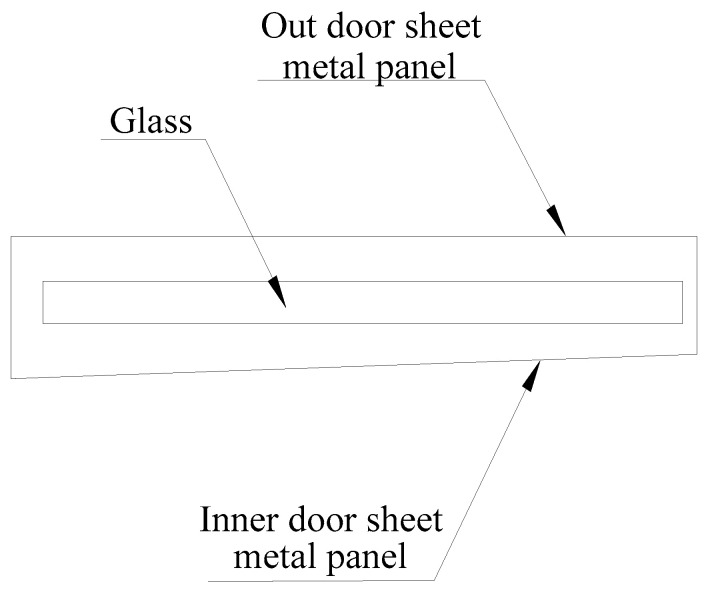
Simplified view of automobile window.

Because the top view of the automobile window looks very much like a letter box, as shown in Figure 9. In the following instructions, a “letter box” is used to represent the structural diagram of the automobile window and the size of the letter box indicates the distance between the inner and outer sheets metal panel of the automobile.

### 3.1. Definition of the 6 Sigma Method

**Figure 10:**
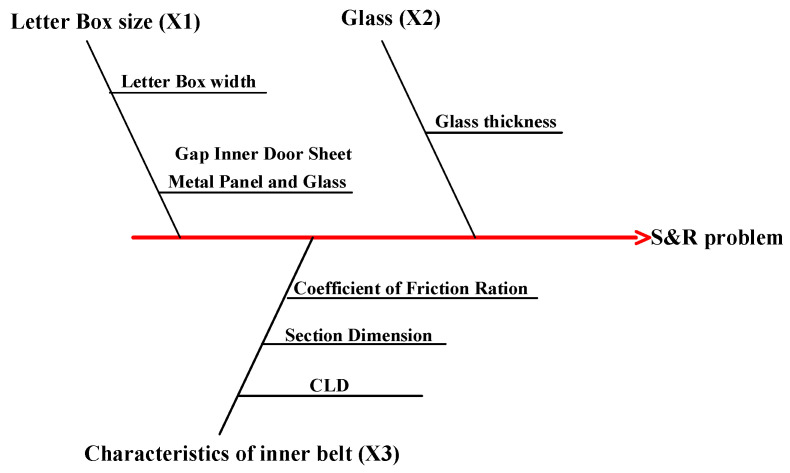
Fishbone diagram of related factors in transverse X. The factors affecting X1, X2 and X3 are listed on the fishbone.

Determine ordinal and transverse coordinates according to the 6 Sigma analysis method. The Y-axis represents the noise when the window is lifting and lowering and the X-axis represents the factors influencing the noise, such as glass thickness, inner belt characteristics and letter box size, as shown in Figure 10.

The key coordinates selected are: X1 for letter box characteristics, X2 for window glass thickness, and X3 for characteristics of inner belt, as shown in Table 2.

The size of the letter box will affect the fit between the window glass and the inner belt, which will cause the friction force to change when the window is lifted or lowered, thus causing the S&R problem. There are strict tolerance standards for the size of automobile window glass. The size of the glass is detected to further determine whether S&R is caused by an inappropriate size of the window glass. The noise is caused by the friction between the window and the inner belt. The factors related to the friction force include the CLD and the flocking position. By changing the CLD, the friction force can be adjusted, and the position of flocking can be adjusted to change the friction coefficient.

### 3.2. Analysis of X1 Using the 6 Sigma Method

#### 3.2.1. Measurement

**Figure 11:**
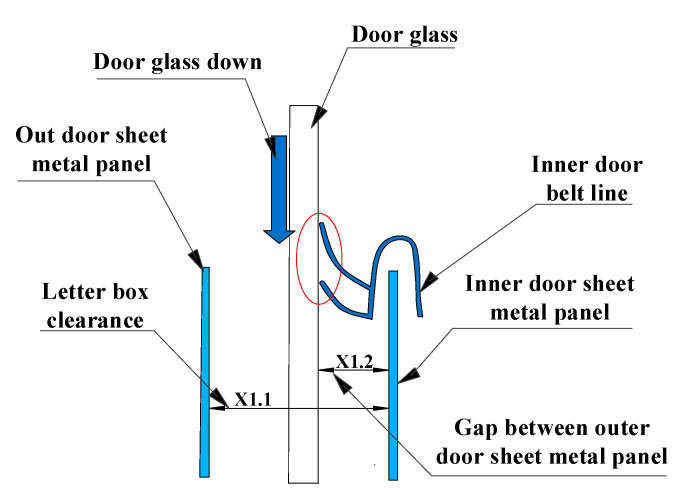
Structure diagram of the window.

**Figure 12:**
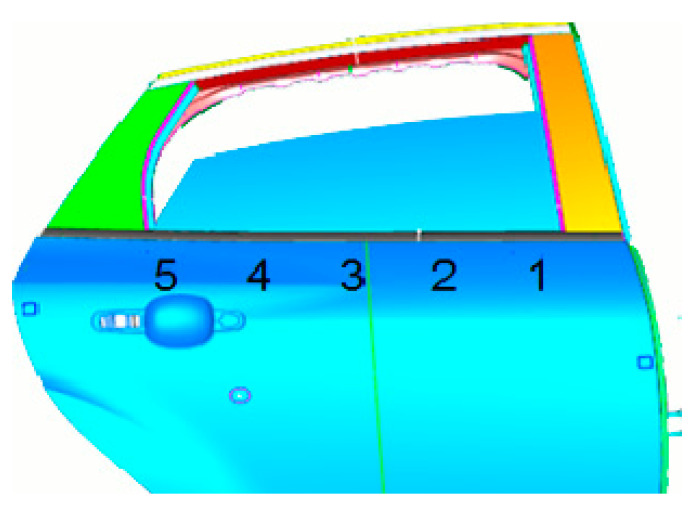
Location map of measuring points for door.

X1.1 represents the width of the automobile window, and X1.2 represents the distance between the window glass and the sheet metal panel in the door, as shown in Figure 11. The letter box of the door of a certain is measured, and the measured points are shown in Figure 12. Measure points 1 to 5 to determine whether X1 meets the production standard.

**Figure 13:**
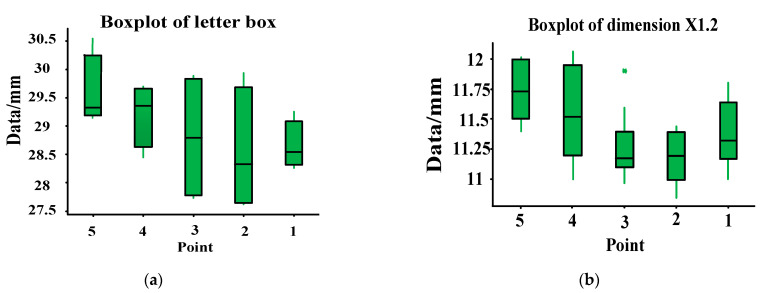
X1 size calculated by Minitab. (**a**) Dimension statistical chart of X1.1; (**b**) dimension statistical chart of X1.2. Fifty percent of the points fall in the green area, the black line in the green area represents the average value, and 20% of the points fall in the upper and lower green solid lines respectively.

Then, the TT Department measured the data of 42 vehicles, and the dimensions of X1.1 and X1.2 were statistically analyzed with Minitab. Minitab is a software package for carrying out the functions of quality management, statistics and data analysis. The size statistics of X1.1 are presented in Figure 13a, and the size statistics of X1.2 are presented in Figure 13b. As can be seen from Figure 13a, the size of the window box tends to increase from point 1 to 5. It can be seen from Figure 13b that the size trend of X1.2 is basically consistent with that of X1.1.

Because the tolerance is required to be controlled to within 2 mm, the data of X1 have problems, and so improvement needs to be carried out before the analysis.

#### 3.2.2. Improvement

**Figure 14:**
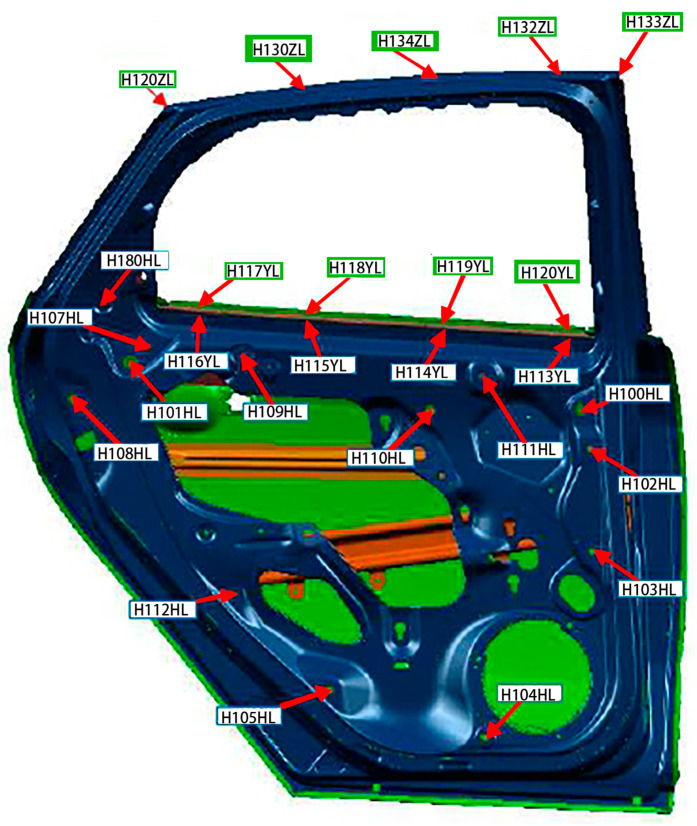
Point measurements of vehicle left front door.

In the previous analysis, we found that the dimensions of X1.1 and X1.2 also have a certain impact on S&R, so we measured the dimensions of the automobile letter box, with the measuring points as shown in Figure 14. The size of X1.1 and X1.2 can be further improved to reduce rattle. The point position of the vehicle left front door was measured, and the data are recorded in Table 3. From the data in Table 3, it can be found that the size of the right door exceeds the limit. The number of testing points and the qualified rate are listed in Table 4.

#### 3.2.3. Analysis

**Figure 15:**
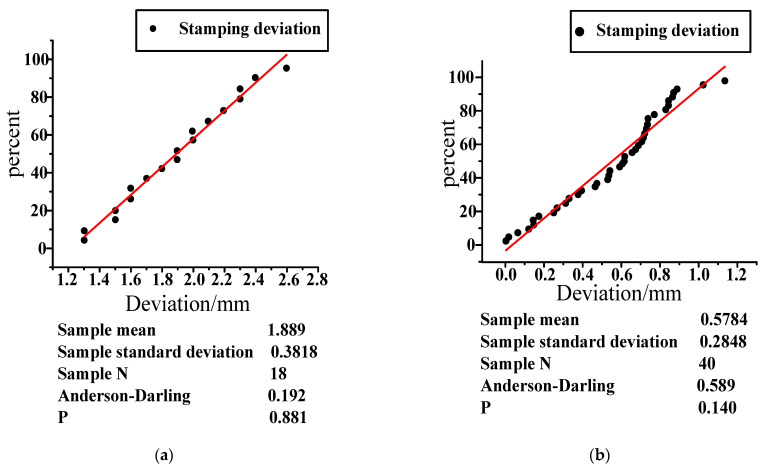
Deviation statistical chart of door position. (**a**) Dimension deviation diagram before improvement; (**b**) improved dimensional deviation diagram. The red line is a standard normal detection line, which is automatically generated by Minitab. The closer the black dot is to the red line, the more the data conforms to the normal distribution.

Then, the dimensions of the stamping workshop are checked, which adjusts the tooling and changes the stamping dimension of the letter box. To accurately calculate the improvement of punching size, the data before punching improvement are presented in Figure 15a with Minitab. The mean value of the sample is 1.889, the number of samples is 18, and the standard deviation is 0.3618. The improved stamping data are presented in Figure 15b with Minitab. The mean value of the sample is 0.5784, the number of samples is 40, and the standard deviation is 0.589. The P-values of both samples are greater than 0.05, which means they are in a normal distribution. T-test for stamping improvement hypothesis:

H0: *μ*1 ≤ *μ*2

H1: *μ*1 > *μ*2

H0 indicates that there is no significant improvement in the processing environment, while H1 indicates a significant improvement in the processing environment.

Both samples belong to small samples and satisfy the normal distribution. The sample data of the two samples are independent from each other, so the number of samples can be different. Because:

(1) $$t=\frac{\bar{X_{1}}-\bar{X_{2}}}{\sqrt{\frac{\left(n_{1}-1\right)S_{1}^{2}+\left(n_{2}-1\right)S_{2}^{2}}{n_{1}+n_{2}-2}\left(\frac{1}{n_{1}}+\frac{1}{n_{2}}\right)}}=6.74667$$

where $\bar{X_{1}}$ is the sample mean in Figure 15a; $\bar{X_{2}}$ is the sample mean in Figure 15b; $n_{1}$ is the sample N in Figure 15; $n_{2}$ is the sample N in Figure 15b; $S_{1}$ is the sample standard deviation in Figure 15a; $S_{2}$ is the sample standard deviation in Figure 15b. Because:

(2) $$t_{\alpha /56}=2.003$$

where α is 56, because $n_{1}+n_{2}-2=56$. As indicated by the t-test checklist:

Because:

$$t>t_{\alpha /56}$$

Therefore, reject the hypothesis of H0 and select H1. Under the level of a = 0.05, the sample size accuracy is significantly improved. It meets the requirements of batch production.

#### 3.2.4. Control (Verification)

**Figure 16:**
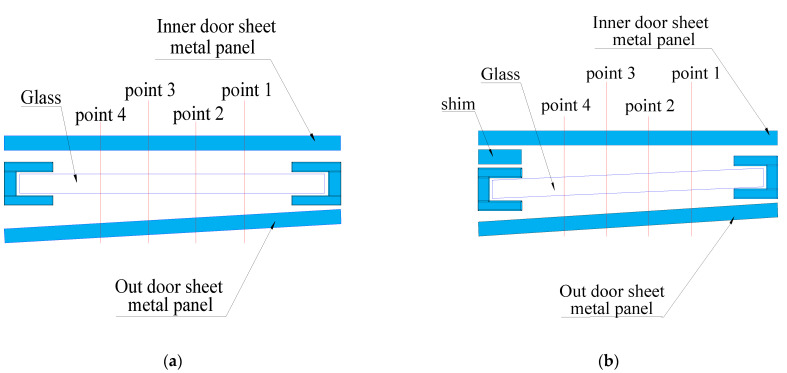
Window structure diagram before and after adding gasket. (**a**) Diagram of window without shim; (**b**) Diagram of window with shim.

**Figure 17:**
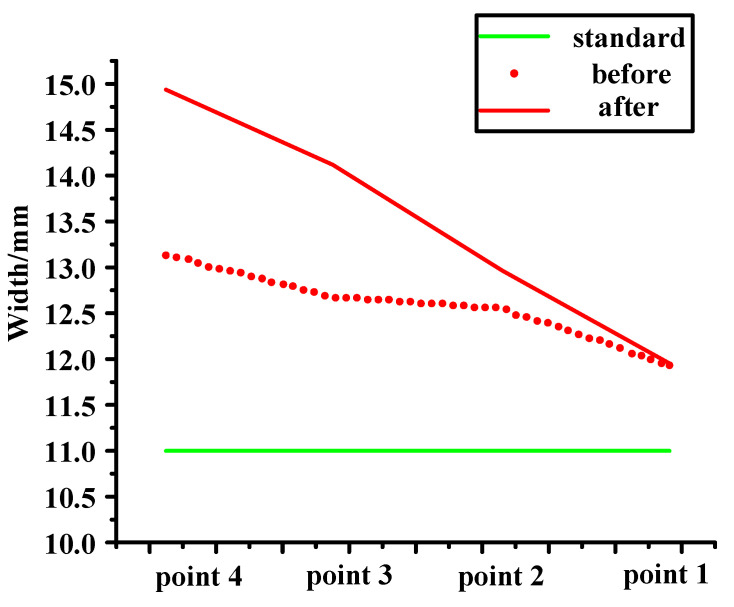
Original and add 1.4 mm.

The position of the vehicle glass is changed, that is to say, the size of X1.2 is changed. The position before the change is shown in Figure 16a. As shown in Figure 15, a 1.4 mm washer is added to the inside of the window glass, so that the size of X1.2 will change. The size of X1.2 before and after the change is presented in Figure 17. It can be seen from the figure that the closer the point is to the shim, the greater the decreasing trend of X1.2. It is found that the noise can be reduced by reducing the distance of X1.2, but the rattle noise still exists. Therefore, X1 is not the key factor in noise generation. In other words, improving X1 to reduce the effect of rattle noise is not obvious. To reduce the S&R, we need to further explore other factors.

### 3.3. Analysis of X2 Using the 6 Sigma Method

#### 3.3.1. Measurement

**Figure 18:**
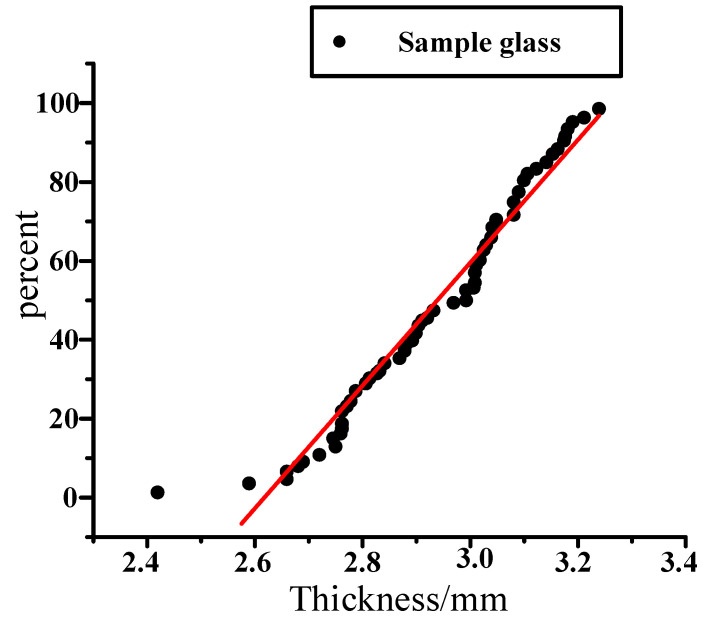
Sample data of window glass.

X2 represents the thickness of the glass. The manufacturing of automobile glass is extremely complicated and must conform to certain standards. We take a group of 60 glass samples to test, and measure the thickness of automobile glass. Minitab was used to create statistics and draw the scatter diagram shown in Figure 18. It can be seen from the figure that the thickness of the glass is basically distributed around the red straight line. This indicates that the thickness of the glass basically conforms to the normal distribution. The graph after statistical analysis with Minitab is shown in Figure 18.

#### 3.3.2. Analysis

According to the data analysis of the 60 glasses, the glass in the sample obeys the normal distribution, and the error is within the allowable range; sample *N* = 60. It is considered that sample mean = overall mean (μ). Overall standard deviation σ is expressed by:

(3) $$\sigma =\sqrt{\frac{\sum_{i=1}^{N}{\left(x_{i}-\mu \right)}^{2}}{N}}=0.186$$

where $x_{i}$ is the thickness of each glass, *N* is the number of the population, and μ is the overall mean. Dispersion Cp is expressed by:

(4) $$Cp=\frac{\left|USL-LSL\right|}{6\sigma }=2.24$$

where USL is the upper limit of deviation and LSL is the lower limit of deviation. The extent to which the mean approaches the lower specification limit Cpl is expressed by:

(5) $$Cpl=\frac{\mu -LSL}{3\sigma }=2.17$$

where μ is overall mean. The extent to which the mean approaches the upper limit of the specification Cpu is expressed by:

(6) $$Cpu=\frac{USL-\mu }{3\sigma }=2.31$$

Comprehensive consideration of intermediate degree and dispersion degree Cpk is expressed by:

(7) Cpk=min(Cpu,Cpl)=2.17

When Cp > 2.0, it indicates that the process is excellent, and when Cpk > 1.5, it indicates that the working condition is excellent.

Sample standard deviation *S* is expressed by:

(8) $$\sigma =\sqrt{\frac{\sum_{i=1}^{N}{\left(x_{i}-\bar{x}\right)}^{2}}{N-1}}=0.178$$

We can calculate the performance indices of process (*PP*), the overall performance capability of a process (*PPK*), and other data according to *N* and *S*. *PPM* is parts per million. Because *PPM* > *USL* and *PPM* < *LSL* are both zero, that is to say, there are 0 unqualified parts in 1 million products, all products are qualified.

**Figure 19:**
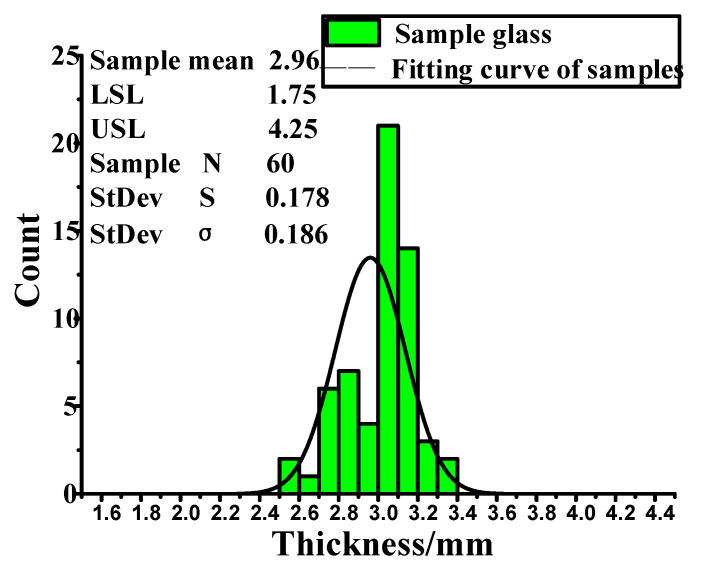
Sample analysis diagram of window glass.

The normal distribution of glass thickness distribution is shown in Figure 19. There is no problem with the size analysis of X2, so there is no follow-up step. The thickness of glass (X2) is not the core of the problem. With improved glass thickness, the effect is not obvious.

### 3.4. Analysis of X3 Using the 6 Sigma Method

#### 3.4.1. Measurement

**Figure 20:**
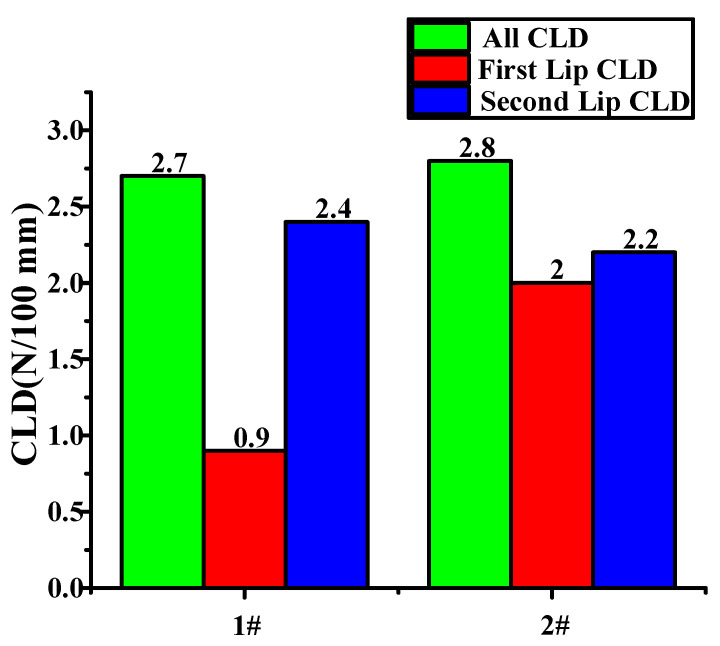
CLD diagram of inner belt.

X3 represents the characteristics of the inner belt, where X3.1 represents the CLD of the inner belt, and X3.2 represents the height of the second lips of the inner belt and the wrapping position of the flocking. The inner belt has flocking cover at the place at which it comes into contact with the glass. The lifting movement of the glass leads to deformation of the inner belt, resulting in no flocking cover at the place where the inner belt comes into contact with the glass. The position of the flocking cover can be seen in Appendix A. Figure 20 shows the CLD of the inner belt of the front left window glass and the inner belt of the rear left window glass. According to the standards for a given automobile, the maximum of internal water shear is 9 N/100 mm, and the maximum extraction load is 3 N/100 mm. However, the CLD of the first and second lips is not specified.

#### 3.4.2. Analysis

The S&R of the automobile’s glass is only present when the window is down; there is a “squeak” sound when the window is down. With the use of detection technology, it was determined that the squeak is produced between the glass and the 2nd lips of the inner belt. The inner belt was removed from the problem vehicle, and the thickness of the glass, the distance between the inner and outer sheet metal, and the characteristics of the inner belt were measured. According to the actual size required to establish a finite element model, finite element analysis software was used to simulate the automobile window down process, to analyze the causes of the automobile squeak.

**Figure 21:**
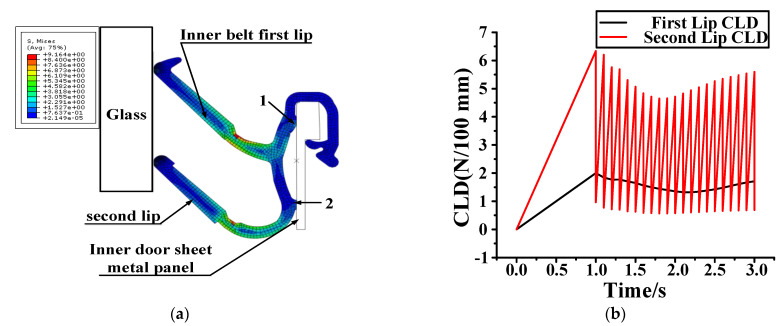
Simulation analysis. (**a**) Pressure load of automobile window; (**b**) inner belt of falling glass.

Compared with the inner belt, the hardness and stiffness of the glass and the inner door sheet metal are larger, so the glass and the inner door sheet metal are set as analytical rigid bodies. There are 28 grids in each area where the inner belt rubs against the glass. The part far away from the glass is sparse, and the total number of cells in the grid is 1627. The inner door sheet metal is fixed, and the inner door metal plate and the inner belt fit together at points 1 and 2. For the need of function, there is a certain preload between the inner belt and the glass, the glass drops at a certain speed (20 mm/s), and the friction coefficient between the glass and the inner belt is 0.4. The glass, inner belt and inner door metal plate are assembled according to their actual size. The movement of the glass when it descends is simulated, and the load on the first and second lips of the inner belt is analyzed, and is reported in Figure 21. The total process lasts for three seconds. The first second is the loading process of the inner belt load. Figure 21a shows the pressure load at the end of the first second. The second and third seconds are the descending process of the internal water shear window glass. In this process, the inner belt rubs against the window glass. The generated friction load is recorded in Figure 21b, from which the pressure change when the glass and the inner belt rub together can be clearly seen. The CLD of the first lip of the inner belt is relatively small, and the change is relatively stable. However, the CLD of the second lip of the inner belt is relatively large, and the change of the load is very unstable in the process of the glass falling, resulting in a serrated load change diagram, which shows that there is a stick–slip effect between the inner belt and the glass. This is the cause of the S&R.

#### 3.4.3. Improvement

**Figure 22:**
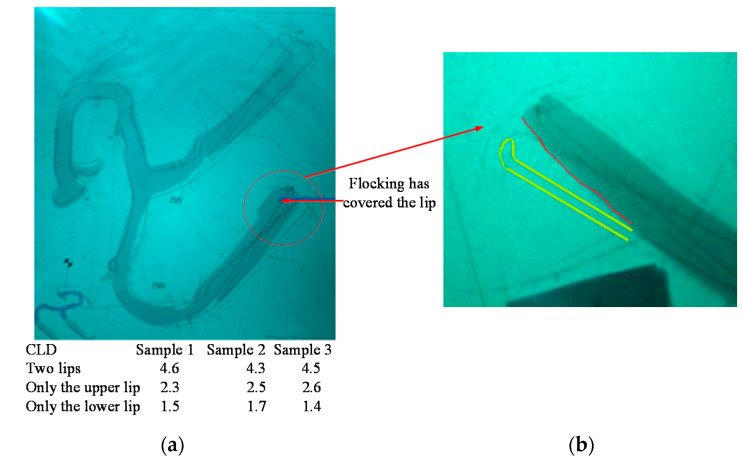
Inner belt test sample in automobile. (**a**) Inner belt sample; (**b**) improved sample of inner belt.

Experiments on these two inner belts revealed that the noise was caused by friction between the 2nd lip of the inner belt and the glass. This may be caused by the CLD of the inner belt, or it may be caused by the improper flocking and coating of the 2nd lip of the inner belt. Firstly, the CLD of the second lip was decreased. To ensure that the overall CLD met the requirements, the CLD of the first lip was increased. The inner belts of the front window and the rear window of the automobile were taken for improvement, and three pairs of samples were taken for the experiment, as shown in Figure 22a.

#### 3.4.4. Control (Verification)

**Figure 23:**
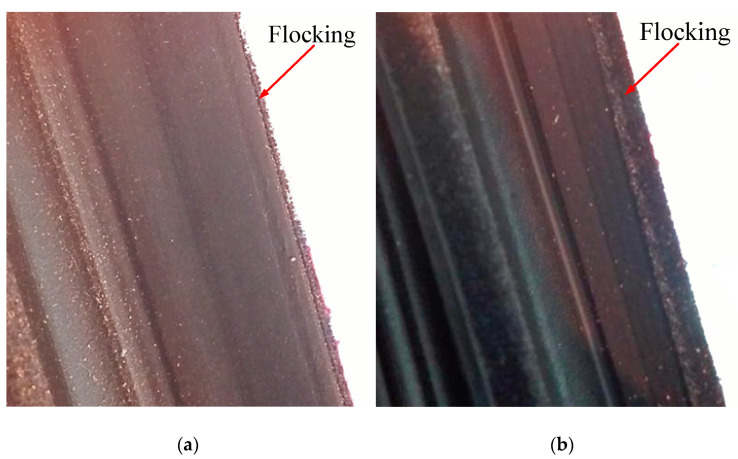
Flocking location map of inner belt. (**a**) Flocking position before improvement; (**b**) improved flocking location.

As there is no flocking coating at the point of friction between the 2nd lip of the inner belt and the glass, the height of the second lips was raised appropriately and the flocking position was changed. The improved position of the second lip is shown in Figure 22b. Then, the flocking position was changed so that the window has a flocking coating at the position where the window rubs against the inner belt when the glass is both down and up. The flocking before improvement is shown in Figure 23a, and the flocking after improvement is shown in Figure 23b. No S&R was observed in the test sample.

**Figure 24:**
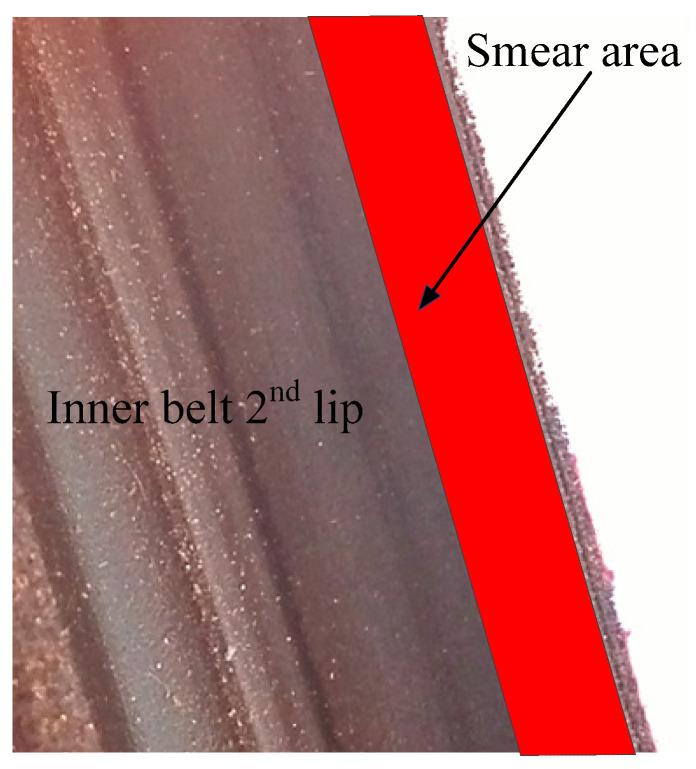
Smear area. The coating is MR3-800.

For vehicles delivered from the factory, the coating MR3-800 can be applied on the second lip of the inner belt. MR3-800 is shown in Figure 6. MR3-800 has a very good effect on reducing friction, and can effectively reduce the friction coefficient. The red area of Figure 24 is the area where the coating MR3-800 should be evenly applied. There was an S&R problem in the inner belt, which was verified by experiments after MR3-800 coating. The effect of this method is tested in the follow-up experiments. The repeated experiments on 62 automobiles show that the effect of this method is excellent. The tracking investigation on the follow-up vehicles shows that the effect can be maintained for about three months.

## 4. Finite Element Analysis and Experimental Verification

### 4.1. Finite Element Analysis

In ABAQUS, numerical simulation is used to further analyze the characteristics of the inner belt. Because 3D modeling takes a lot of time, the model is simplified as much as possible, and it is simplified into a two-dimensional plane strain finite element model. Persson and Popov [20,21] performed some research on dimension reduction and the contact problem. Due to the large difference in stiffness between the glass and the inner belt, and in order to reduce the calculation time, the glass is set as an analytical rigid bod. The internal water shear is set as an ordinary variable body. The Young’s modulus *E* is 4.5 MPa, the density ρ is 0.78 g/cm^3^, the Poisson’s coefficient *v* is 0.4, and the friction coefficient is 0. The motion is divided into two parts: static and implicit dynamics.

**Figure 25:**
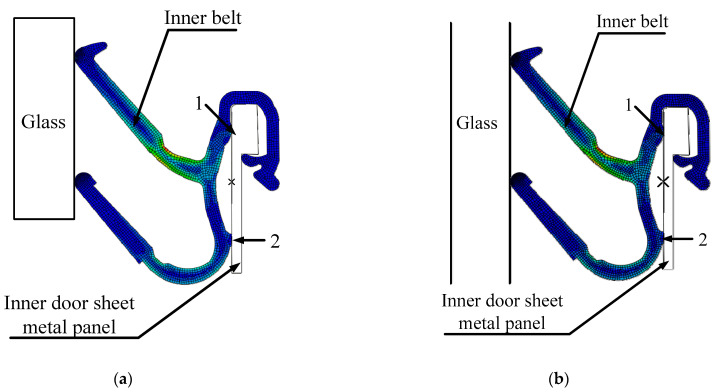
Load carrying cloud diagram of inner belt. (**a**) Stress load at 1 s; (**b**) stress load at 3 s.

**Figure 26:**
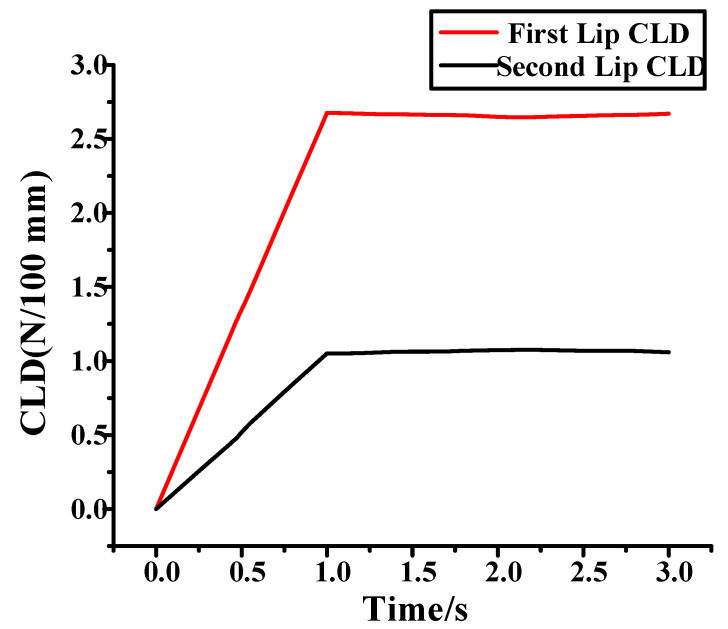
Change image of inner belt CLD (frictionless coefficient).

The first step is to preload the inner belt load. A depiction of the pressure load following preloading is shown in Figure 25a. Because of the improvement of the inner belt structure, the CLD of the 2nd lip becomes smaller after the inner belt is preloaded. The second step is the process of the glass moving downward, the falling speed is 20 mm/s, the simulation time of the falling process is 2 s, and the pressure load following this is shown in Figure 25b. Since rattle noise is caused by the collision between the second lip of the inner belt and the glass, Figure 26 shows the change of the CLD between the inner belt and the glass. On the basis of the numerical simulation, it is found that reducing the CLD of the second lip and the friction coefficient of the inner belt lip can effectively eliminate the rattle noise and completely solve the problem of rattle. The method for reducing the CLD of the second lip is to increase the height of the second lip.

#### 4.1.1. Setup of Coulomb Friction Model

**Figure 27:**
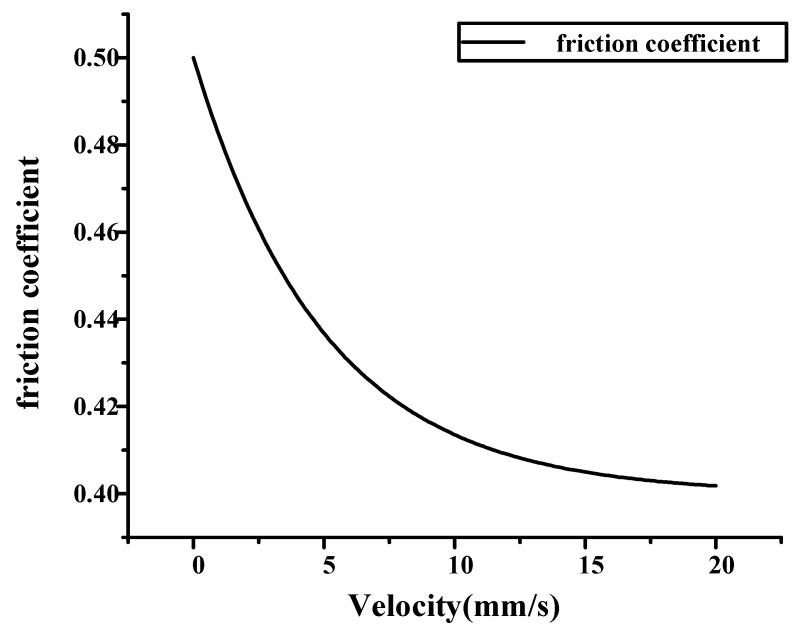
Friction model with exponential decay.

ABAQUS software provides a friction model that directly specifies static friction coefficient and dynamic friction coefficient. In this model, it is assumed that the static friction coefficient decreases exponentially over the sliding velocity towards the dynamic friction coefficient. The calculation formula for the friction coefficient μ is as follows:

(9) $$\mu =\mu_{k}+\left(\mu_{s}-\mu_{k}\right)e^{-d\dot{\gamma }}$$

where $\mu_{k}$ is the dynamic friction coefficient; $\mu_{s}$ is the static friction coefficient; *d* is the attenuation coefficient; and $\dot{\gamma }$ is the equivalent slip velocity. To verify the load characteristics under high friction coefficient, $\mu_{k}$ is set to 0.4, $\mu_{s}$ is set to 0.5, and *d* is set to 0.2. An exponential decay model of the friction coefficient with relative sliding velocity is thus obtained, as shown in Figure 27.

#### 4.1.2. Implicit Dynamic Analysis

In the process of nonlinear analysis, ABAQUS is not able to solve the problem simply by solving a set of equations. The problem must be solved step by step in the form of incremental equations by gradually applying the load boundary. Each incremental step in the implicit method is related to the setting of the load and boundary. When the load on the structure changes with time, the stiffness of the structure will change with the deformation. When the structure has a large deformation under the external load, the material nonlinearity, geometric nonlinearity and boundary condition nonlinearity should be considered at the same time. The Newton Raphson algorithm is used to solve nonlinear equations in ABAQUS/standard module. In the nonlinear analysis, the equation for iterative balance control is mainly carried out through the semi-incremental step residual. Consider the external forces acting on the structure *F*, internal nodal forces *I*, and D’Alembert $M\ddot{u}$. When the object is in equilibrium, the force on the node should be 0. Therefore, the basic basis for judging the balance of the semi-incremental step residual is the internal node force *I* at the semi-incremental step. D’Alembert $M\ddot{u}$ and external forces *F* have to be balanced.

In the implicit Newmark method for the process of equilibrium iteration of time integration, the acceleration Δ*t* is assumed to vary linearly.

(10) $$M\ddot{u}+I-F=0$$

The displacement vector and velocity vector of the system at the half increment step *t* + Δ*t*/2 can be obtained using the integral algorithm.

(11) $${\ddot{u}}_{t+\Delta t/2}=\frac{1}{2}\times \left({\ddot{u}}_{t+\Delta t}+{\ddot{u}}_{t}\right)$$

(12) $$u_{t+\Delta t/2}=u_{t}+\frac{1}{8}\times \Delta u+\frac{3}{8}\times \Delta t\times {\ddot{u}}_{t}+\frac{\Delta t^{2}}{16}\times \Delta {\ddot{u}}_{t}$$

The external force of the half increment step can be approximately determined using Formula (13).

(13) $$F_{t+\Delta t/2}=F+\frac{1}{2}\times \Delta F$$

ABAQUS/standard uses the structure configuration u at a certain time. Initial stiffness K and ΔF are used to calculate the displacement correction of the structure $C_{t+\Delta t/2}$. Then, the configuration of the structure is updated to $u_{t+\Delta t/2}$ by $C_{t+\Delta t/2}$. At *t* + Δ*t*/2, the D-value of applied external load is $F_{t+\Delta t/2}$, internal force is $I_{t+\Delta t/2}$ and D’Alembert $M{\ddot{u}}_{t+\Delta t/2}$ is the so-called half incremental step residual.

(14) $$R_{t+\Delta t/2}=M{\ddot{u}}_{t+\Delta t/2}+I_{t+\Delta t/2}-F_{t+\Delta t/2}$$

If the value of $R_{t+\Delta t/2}$ for each degree of freedom of the model is 0 and the calculated point is on the load displacement curve, the structure is in equilibrium.

#### 4.1.3. Grid Division

Because the inner door metal plate and glass are set as analytical rigid bodies, there is no need to divide the mesh. It is only necessary to divide the mesh of the inner belt. The mesh of the inner belt is mainly divided into a quadrilateral free mesh, using the advanced algorithm in non-conforming mode.

**Figure 28:**
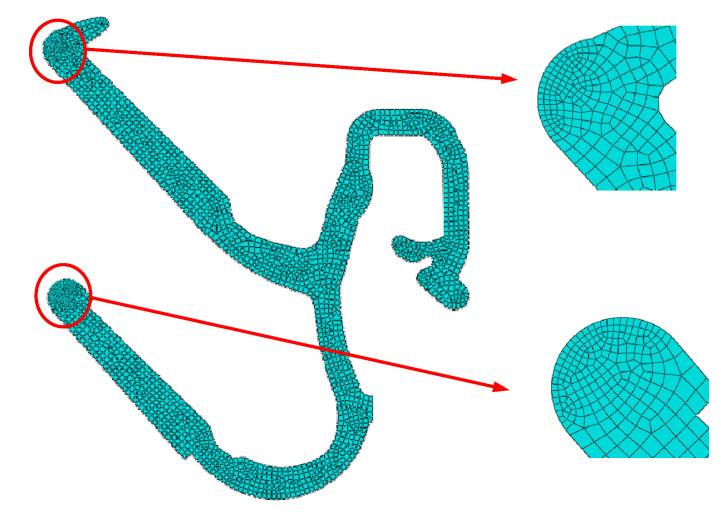
Inner belt mesh generation.

Because the first and second lips of the inner belt are in contact with the glass, the mesh should be divided carefully. The number of the first and second lips should not be less than ten, which is able to meet the requirements of accuracy. The mesh is shown in Figure 28.

#### 4.1.4. Simulation Analysis

**Figure 29:**
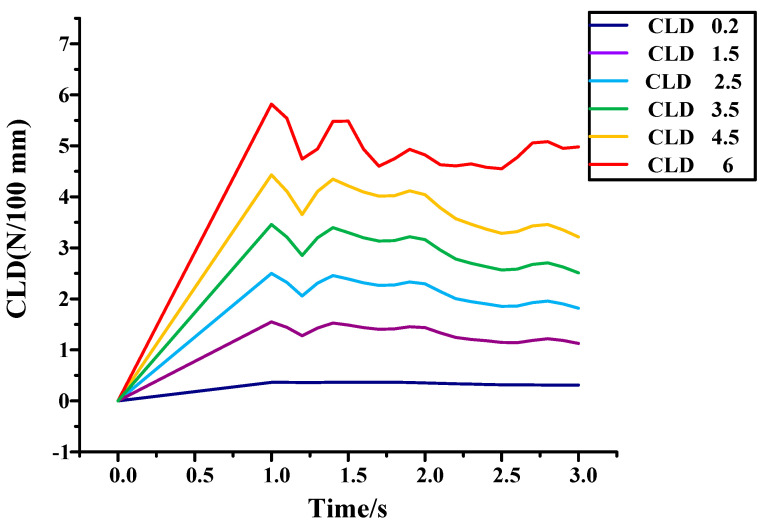
Simulation analysis of different CLD of inner belt.

Because the first lip of the inner belt has two points of support (Figure 21a), the second lip has only one point of support, which has poor stability and low structural stiffness. Therefore, the second lip is simulated separately under different CLD. The results are shown in Figure 29.

It can be seen from the figure that the larger the CLD, the more obvious the instability of the second lip. Due to the instability of the second lip, rattle may occur. Therefore, it is necessary to reduce the excitation to the second lip. When the CLD is below 3 N/100 mm, the instability becomes smaller, and when the CLD is above 4 N/100 mm, the instability becomes more obvious. The internal water will beat the glass like a rattle. When the friction coefficient is less than 0.25, the instability is greatly reduced. Therefore, the CLD of the inner belt should be between 3–9 N/100 mm, and the CLD of the second lip should be less than that of the first lip. A drawing of the improved inner belt is shown in Appendix A.

### 4.2. Real Vehicle Verification

We installed the improved inner belt on the window and performed experiments 50 times in the morning and afternoon, respectively. In the case of low humidity, the problem vehicle was verified by experimental verification, and again there was no S&R. The specific time, humidity and results of the experiment are shown in Table 5. The experimental results show that there is no S&R at low humidity.

Because many of the cars are driven on rainy days, it is necessary to carry out high-humidity experiments. First, water is sprayed on the surface of the automobile; after the surface of the automobile is completely wet, the S&R experiment of moving the window up and down is performed.

VIN17 (26) represents an automobile type. FL is the front left window, FR is the front right window, RL is the rear left window, RR is the rear right window. No indicates no S&R. The problem vehicle was verified in the subsequent high-humidity test. The experimental data are recorded, as shown in Table 6. After the improvement of the problem vehicle, no further problems occurred.

## 5. Conclusions

Aiming to address the S&R problem of descending automobile windows, the key dimensions of automobile windows were improved as follows using the 6 sigma analysis method: (1) the accuracy of the box size of the window was improved; (2) the CLD of the inner belt was 3–9 N/100 mm, and the CLD of the second lip was less than that of the first lip; (3) the position of the flocking coating of the 2nd lips of the inner belt was changed so that the window will always rub against the flocking during the lifting process. Through the improvement of the above three points, the S&R problem of window lifting and lowering was completely solved.

## Figures and Tables

**Table 1:** Rubber properties.

Mechanical Property	Numerical Value
Young’s modulus	4.5 MPa
tensile strength	9~20.8 MPa
Shore hardness	40~90

**Table 2:** Control factors.

Subcode	Control Factor
X1.1	Letter box size
X1.2	Distance between glass and inner sheet metal panel
X2	Thickness of glass
X3.1	CLD of inner belt
X3.2	Flocking position of inner belt 2nd lips

**Table 3:** Window point measurement. (The default unit is mm. The date of data measurement is September 29, November 3, December 2 and December 4. More than 2 mm is marked with red background. The datum plane of the coordinate is the horizontal ground. The data in the table present the difference between the measured value and the reference value.).

Point Position	Coordinates/ Distance	9.29	11.3	11.3	12.2	12.2	12.4	12.4
H113YL	764.04	0.6	0.1	0.1	−0.2	−0.4	−0.3	−0.2
H113YL H120YL	24.95	−1.2	1.3	1.1	1.4	1.4	1.3	1.1
H114YL	757.93	0.5	0.5	0.6	−0.3	−0.2	−0.2	−0.2
H114YL H119YL	24.64	−0.7	1.5	1.3	2.3	1.9	2.1	2.0
H115YL	749.35	0.5	0.2	0.3	−0.4	−0.8	−0.3	−0.3
H115YL H118YL	24.8	0.3	2.2	1.9	2.4	2.6	2.3	2.0
H116YL	741.95	0.5	0.7	0.9	0.2	−0.1	0.3	0.5
H116YL H117YL	23.83	0.5	1.7	1.5	1.6	1.8	1.6	1.3
H117YL	765.43		2.4	2.3	1.8	1.7	1.9	1.8
H118YL	774.15		2.4	2.2	2.0	1.8	2.0	1.8
H119YL	782.57		2.0	1.9	2.1	1.7	1.9	1.7
H120YL	788.99		1.4	1.3	1.2	1.0	0.9	0.9

**Table 4:** Window point analysis table.

Date	9.29	11.3	11.3	12.2	12.2	12.4	12.4
(Pass rate) Tolerance 1.5 mm	95.8%	95.1%	95.1%	94.2%	94%	94.2%	95.3%
Total points (number)	118	634	634	634	634	634	634
Unqualified points (number)	5	38	39	43	46	41	36

**Table 5:** Validation results.

Date	Humidity	Test Frequency	Test Result
10.31 PM	36%	50	No S&R
11.1 AM AND PM	77%	50	No S&R
11.2 PM	44%	50	No S&R
11.3 AM	44%	50	No S&R
11.3 PM	29%	50	No S&R
11.4 AM	56%	50	No S&R

**Table 6:** Experimental statistics.

Type		Up/ Below	Up/ Middle	Up/ Top	Down/ Top	Down/ Middle	Down/ Below	Condition	Note
VIN17	FL	no	no	no	no	no	no	Wet	50 times
VIN17	FR	no	no	no	no	no	no	Wet	50 times
VIN17	RL	no	no	no	no	no	no	Wet	50 times
VIN17	RR	no	no	no	no	no	no	Wet	50 times
VIN26	RL	no	no	no	no	no	no	Wet	50 times
VIN26	RR	no	no	no	no	no	no	Wet	50 times

**Figure A1:**
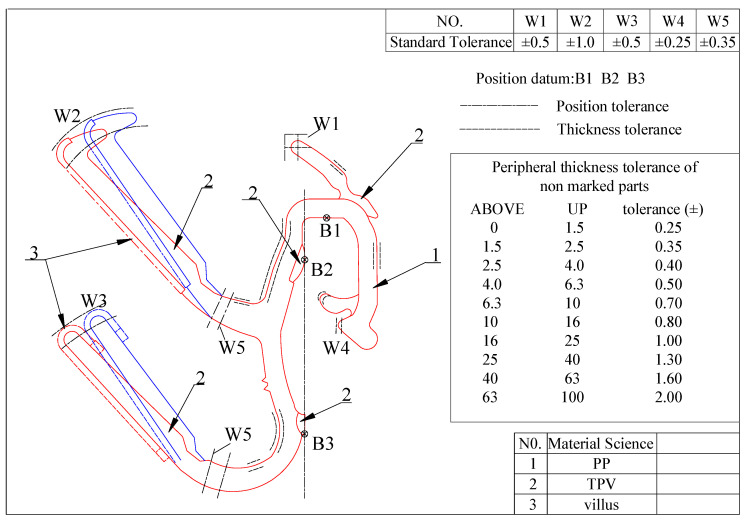
The optimized cross-section of the inner belt of the automobile door.

## Data Availability

The data in this article is true and valid.
